# Epstein-Barr Virus latent membrane protein 1 induces Snail and epithelial–mesenchymal transition in metastatic nasopharyngeal carcinoma

**DOI:** 10.1038/bjc.2011.38

**Published:** 2011-03-08

**Authors:** T Horikawa, T Yoshizaki, S Kondo, M Furukawa, Y Kaizaki, J S Pagano

**Affiliations:** 1 Lineberger Comprehensive Cancer Center, University of North Carolina at Chapel Hill, CB# 7295, Chapel Hill, NC 27599-7295, USA; 2 Department of Otolaryngology, School of Medicine, Kanazawa University, 13-1 Takara-machi, Kanazawa 920-8641, Japan; 3 Department of Pathology, Fukui Prefectural Hospital, 2-8-1 Yotsui, Fukui 910-8526, Japan

**Keywords:** Snail, latent membrane protein 1, epithelial–mesenchymal transition, metastasis, nasopharyngeal carcinoma

## Abstract

**Background:**

Epstein-Barr Virus (EBV)-associated nasopharyngeal carcinoma (NPC) is distinctive among head-and-neck cancers in its undifferentiated histopathology and highly metastatic character. We have recently investigated the involvement of epithelial–mesenchymal transition (EMT) in NPC. In a previous study, we found a close association of expression of LMP1, the principal EBV oncoprotein, with expression of Twist and induction of EMT.

**Methods:**

We analysed expression of Snail in 41 NPC tissues by immunohistochemistry. The role of Twist as well as Snail in EMT of NPC was investigated by using NP69SV40T human nasopharyngeal cells.

**Results:**

In NPC tissues, overexpression of Snail is associated with expression of LMP1 in carcinomatous cells. In addition, expression of Snail positively correlated with metastasis and independently correlated inversely with expression of E-cadherin. Expression of Twist had no association with expression of E-cadherin. Further, in a human nasopharyngeal cell line, LMP1 induces EMT and its associated cellular motility and invasiveness. Expression of Snail is induced by LMP1 in these cells, and small hairpin RNA (shRNA) to Snail reversed the cellular changes. By contrast, Twist did not produce EMT in these nasopharyngeal cells.

**Conclusions:**

This study strengthens the association of EMT with the metastatic behaviour of NPC. These results suggest that induction of Snail by the EBV oncoprotein LMP1 has a pivotal role in EMT in NPC.

Evidence has been growing that metastatic carcinoma cells activate the dormant epithelial–mesenchymal transition (EMT) programme, which promotes cell migration, invasion and metastasis (Yang and Weinberg, 2008; Thiery *et al*, 2009). Epithelial–mesenchymal transition is a process whereby epithelial cells lose cell–cell contacts and undergo remodelling of the cytoskeleton, thus resulting in a migratory phenotype. Epithelial–mesenchymal transition was first recognised in embryogenesis, and recently it has been implicated in several metastatic malignancies including nasopharyngeal carcinoma (NPC) (Yang *et al*, 2004; Rees *et al*, 2006; Horikawa *et al*, 2007; Yang and Weinberg, 2008; Thiery *et al*, 2009). Moreover, recent studies of EMT have fuelled a change in paradigm on occurrence of cancer metastases from the late metastasis theory to the early metastasis model (Ansieau *et al*, 2008; Klein, 2008; Mani *et al*, 2008; Sánchez-García, 2009).

Nasopharyngeal carcinoma, a cancer with high incidence in southeast Asia, is known for its highly metastatic character in early stages of the disease (Lo *et al*, 2004; Wei and Sham, 2005; Lo *et al*, 2006). Also, no human carcinoma is as consistently associated with Epstein-Barr Virus (EBV) as NPC, and EBV, the first human tumour virus, is intimately associated with its oncogenesis (Raab-Traub, 2002; Lo *et al*, 2004; Pagano *et al*, 2004; Wei and Sham, 2005; Lo *et al*, 2006; Pagano, 2010). The present study derives from the hypothesis that the distinctive undifferentiated histopathology and early metastatic character of NPC might point to the occurrence of EMT induced by EBV in the genesis of this tumour. Tumour cells of undifferentiated NPC exhibit EMT-like phenotypes: irregular morphology with loss of tight intracellular adhesion and epithelial structures; diffusely infiltrative growth histologically; and metastasis-proneness from early phases of the disease (Marks *et al*, 1998; Wei and Sham, 2005).

Latent membrane protein 1 (LMP1) is the principal EBV oncoprotein and required for immortalisation of B-lymphocytes (Kaye *et al*, 1995). LMP1 acts as a constitutively active EGF receptor-like, but ligand-less molecule and is considered to contribute to initiation of EBV-related malignancies (Knecht *et al*, 2001). It is detected in at least 70% of NPC at the protein level and in virtually all at the RNA level (Pathmanathan *et al*, 1995). Besides its aetiologic role, LMP1 has been implicated in tumour progression (Yoshizaki *et al*, 1998, 2005; Lo *et al*, 2006). The propensity to metastasis of EBV-related malignancies corresponds with the expression of LMP1 (Pagano, 2009). Nasopharyngeal carcinomas with high levels of LMP1 tend to be more metastatic than those with low levels (Horikawa *et al*, 2000, 2001; Sarac *et al*, 2001). Further, underpinning these associations are numerous observations that LMP1 can upregulate a constellation of metastasis-related factors (Takeshita *et al*, 1999; Murono *et al*, 2000, 2001; Yoshizaki *et al*, 2001; Wakisaka *et al*, 2002, 2004; Wakisaka and Pagano, 2003; Kondo *et al*, 2005, 2006a, 2006b, 2007; Endo *et al*, 2009).

Twist, Snail, SIP1 and Slug are known as prominent E-cadherin repressors and EMT regulators (Yang and Weinberg, 2008). Different levels of expression and roles for these regulators have been reported in a variety of invasive carcinomas. Snail, a zinc-finger transcription factor, is a representative regulator of EMT during embryogenesis (Barberà *et al*, 2004; Bachelder *et al*, 2005). More importantly, the expression of Snail is upregulated in invasive types of carcinoma cell lines (Cano *et al*, 2000) and in invasive human carcinomas, including undifferentiated breast carcinomas (Blanco *et al*, 2002) and hepatocellular carcinomas (Yang *et al*, 2009). Snail increases aggressiveness of experimentally induced breast tumours, and its overexpression is associated with recurrence of human breast cancer (Moody *et al*, 2005). However, the pathogenesis of EMT and upstream signalling pathways for EMT regulators in malignancies are still not well understood.

In a recent study, we reported the association of EMT with metastatic NPC. We found that LMP1 induces EMT through Twist in the Madin-Darby Canine-Kidney (MDCK) cell line and that Twist is associated with LMP1 and metastasis in NPC (Horikawa *et al*, 2007). Here, we explore further the involvement of EBV and EMT in metastasis and NPC in relation to the EMT regulators, Snail, SIP1 and Slug. Instead of canine-kidney cells, we exploit a newly established human nasopharyngeal cell line for this purpose. Interestingly although the expression of Snail is also associated with LMP1 and with metastasis, the level of Snail, but not Twist, inversely correlated with the level of E-cadherin in NPC. Snail appears to be essential for induction of EMT by LMP1 as shown when its expression is inhibited by Snail shRNA in the human nasopharyngeal cells. Twist did not produce EMT in these nasopharyngeal cells. These results suggest that Snail has a pivotal role in EMT and metastasis in NPC.

## Materials and methods

### NPC tissues

A total of 41 tissues from paraffin-embedded NPC specimens were from Fukui Prefectural Hospital, Fukui, Japan. Tissues from 37 cases from the same set used in our previous study (Horikawa *et al*, 2007) plus four additional cases were analysed.

### Immunohistochemical analysis

Immunohistochemistry was done as described before (Horikawa *et al*, 2000, 2001, 2007). Primary antibodies used were: mouse LMP1 monoclonal antibody from DAKO (Grostrup, Denmark), goat Snail, rabbit SIP1, rabbit Slug and rabbit Twist polyclonal antibodies, and mouse E-cadherin monoclonal antibody from Santa Cruz Biotechnology (Santa Cruz, CA, USA). Two examiners independently selected two representative fields of more than 200 tumour cells from each tissue specimen and counted both stained and total number of tumour cells without knowledge of the clinical data. Average percentages of stained cells were used to calculate the LMP1, Snail, SIP1, Slug, Twist and E-cadherin expression scores, as in our previous study (Horikawa *et al*, 2000, 2001, 2007, 2008). Staining was repeated at least twice in sequential sections to assess reproducibility. The expression scores of LMP1 and Twist for 37 cases were taken from our previous results (Horikawa *et al*, 2007) and analyzed again to derive a score that included the additional four cases. Staining of LMP1 and Twist for the previous 37 cases was done again to confirm reproducibility.

### Statistical analysis

Pearson’s correlation coefficient was used to detect correlations among the expression scores of each protein analyzed in NPC. The expression score of each protein in relation to clinical data was analyzed with the Mann–Whitney *U*-test. The differences in wound migration and invasion indices between NP69SV40T cell clones were analyzed by the paired *t*-test.

### Cell cultures

NP69SV40T immortalised human nasopharyngeal epithelial cells were the kind gift of Dr Sai Wah Tsao, University of Hong Kong, Hong Kong, China (Tsao *et al*, 2002). Cells were maintained as described (Tsao *et al*, 2002).

### Plasmids and small hairpin RNA

PcDNA3-based LMP1 plasmid has been described (Yoshizaki *et al*, 1998). The pSUPER-Snail-shRNA and pSUPER-control-shRNA were kind gifts from Dr Gerhard Christofori (University of Basel, Basel, Switzerland) and have been described previously (Grotegut *et al*, 2006).

### Transient and stable transfections

Cells were transfected with FUGENE6 transfection reagent (Roche Diagnostics, Indianapolis, IN, USA). Stable cell lines expressing LMP1 were established in the presence of 500 *μ*g ml^−1^ G418.

### Retroviral transduction

Retroviral transduction has been described previously (Moody *et al*, 2005). Snail-shRNA and control-shRNA infected cells were selected with 2 *μ*g ml^−1^ puromycin.

### Western blot analysis

Cell lysates (100 *μ*g) were analyzed for protein levels by western blotting (Wakisaka *et al*, 2002). Primary antibodies for LMP1, Snail and E-cadherin are the same as those used for immunohistochemistry. Primary antibodies: *α*-catenin polyclonal antibody, goat vimentin polyclonal antibody and rabbit N-cadherin polyclonal antibody from Santa Cruz Biotechnology; and mouse *γ*-tubulin monoclonal antibody from Sigma (St Louis, MO, USA).

Reverse-transcriptase polymerase chain reaction (RT–PCR) analysis. RT–PCR for Snail was carried out as described with specific primers as follows: sense, 5-TGCGCGAATCGGCGACCC-3; antisense, 5-CCTAGAGAACCGCTTCCCGCAG-3 (product size, 600 bp) (Elloul *et al*, 2005).

### Invasion assay and cell wound-migration assay

Cell invasiveness was assessed in Biocoat Matrigel Invasion Chambers (Becton Dickinson Labware, Bedford, MA, USA) (Murono *et al*, 2000; Horikawa *et al*, 2007). Cell wound-migration assay was done as described before (Horikawa *et al*, 2007).

Immunofluorescence studies. Procedures were performed as described (Gershburg *et al*, 2004; Horikawa *et al*, 2007). Primary antibodies for Snail, E-cadherin and N-cadherin were the same as used for immunohistochemistry.

## Results

### Snail is overexpressed in NPC tumours

**Figure 1:**
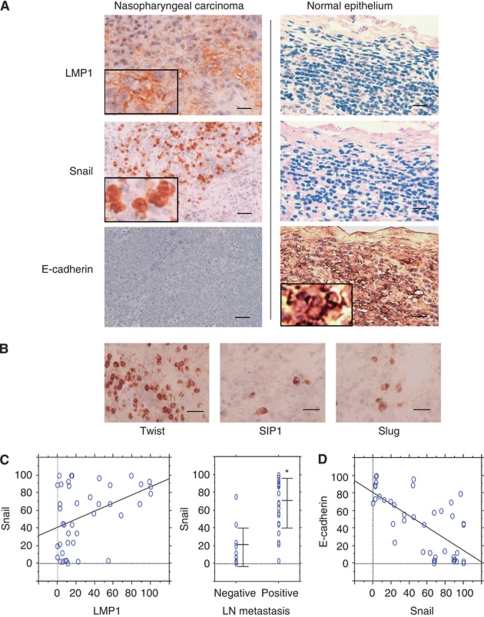
Expression of Snail and LMP1 are associated, and Snail correlates directly with metastasis and inversely with expression of E-cadherin in NPC. (**A**) LMP1 and Snail are overexpressed in NPC tumour cells, but not detected in adjacent normal nasopharyngeal epithelium by immunohistochemistry. Representative results in NPC and in normal nasopharyngeal epithelium are shown. Bars, 50 *μ*m. (**B**) Twist, SIP1 and Slug are expressed in nuclei of tumour cells in NPC; Twist is highly expressed in tumour-cell nests. (**C**) Expression scores of Snail and LMP1 correlated significantly in NPC: Pearson's correlation coefficient, *r*=0.415, *P*=0.0065. Expression levels of Snail in relation to incidence of cervical lymph-node metastasis. ^*^Significance according to Mann–Whitney *U* test, *P*<0.0001. (**D**) Expression of Snail correlates inversely with expression of E-cadherin in NPC (*r*=−0.68, *P*<0.0001).

Overexpression of Twist in NPC was reported recently (Horikawa *et al*, 2007) and is shown again in Figure 1B. Here, we examine expression of representative EMT regulators Snail, SIP1 and Slug by immunohistochemistry in NPC tissue sections. Staining for SIP1 and Slug was detected in the nuclei of tumour cells, but expression was sporadic and at very low levels (Figure 1B). By contrast, Snail was readily detected in the nuclei of tumour cells and was clearly overexpressed in tumour nests (Figure 1A).

### Expression of Snail correlates with expression of LMP1 in NPC and correlates directly with metastasis

We analyzed if there is correlation of expression of Snail, SIP1 or Slug with LMP1 in NPC. Neither the expression of SIP1 nor Slug showed such an association (data not shown), whereas analysis of Snail revealed significant positive correlation with expression of LMP1 (*r*=0.415, *P*=0.0065) (Figure 1C). Clinically, regional lymph-node invasion is the most common presenting finding in NPC (Lo *et al*, 2004), mirroring its highly metastatic character. NPC often shows marked cervical lymph-node metastasis before forming a mass in the primary site. In 29 cases with cervical lymph-node metastasis, the Snail expression score was 69.5±27.1 (mean±s.d.). In 12 cases without metastasis, the score was 16.4±22.5. Thus, Snail expression in metastasis-positive cases was significantly higher than in metastasis-negative cases (*P*<0.0001) (Figure 1C). Expression of Twist also significantly correlated with cervical lymph-node metastasis in the 41 cases studied (*P*=0.001).

### Expression of Snail correlates independently inversely with expression of E-cadherin

Having found close association of Snail as well as Twist with metastasis of NPC, we next probed for evidence of EMT in NPC tissue. Snail and Twist repress E-cadherin through binding to E-boxes in the E-cadherin promoter and induce EMT (Yang and Weinberg, 2008). Downregulation of E-cadherin is a hallmark of EMT (Thiery *et al*, 2009). Therefore, we examined expression of E-cadherin and analysed its association with the EMT regulators in NPC. E-cadherin was regularly detected on membranes of tumour cells by immunohistochemistry. Expression of neither SIP1 nor Slug was associated with expression of E-cadherin (data not shown). Unexpectedly, expression of Twist was not associated with expression of E-cadherin (*r*=−0.22, *P*=0.17), whereas the pattern of expression of Snail appeared to be the inverse of that of E-cadherin. In normal nasopharyngeal epithelium, cells are tightly packed and structured. Here, expression of E-cadherin is highly conserved, and expression of neither LMP1 nor Snail proteins was detected (Figure 1A). By contrast, in NPC tumour nests where undifferentiated tumour cells show disseminative and diffusely organised morphology, LMP1 as well as Snail protein were overexpressed, whereas the expression of E-cadherin was at low levels (Figure 1A). As shown in Figure 1D, the expression of Snail correlated inversely with the expression of E-cadherin in NPC (*r*=−0.68, *P*<0.0001). The results indicate the close association of Snail, but not Twist, with EMT in NPC.

### LMP1 could induce Snail in AdAH nasopharyngeal cells

**Figure 2:**
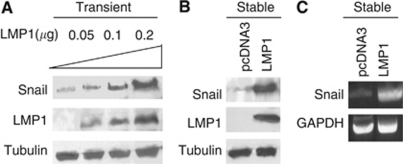
Expression of Snail protein and mRNA is induced by LMP1 in Ad-AH human nasopharyngeal epithelial cells. (**A**) Different amounts of LMP1 expression plasmid were transiently transfected and Snail protein levels were determined by western blotting. (**B**) Expression of Snail is increased in Ad-AH cells stably expressing LMP1. (**C**) Snail mRNA level is upregulated by LMP1. Extracts from the same cells in Figure 2B were analyzed by RT–PCR.

Having found a close association of Snail with LMP1 in NPC, we explored whether LMP1 can induce Snail in the human nasopharyngeal epithelial cell line, AdAH, used for study of LMP1 in previous studies (Yoshizaki *et al*, 2005). In AdAH cells transiently transfected with LMP1, Snail was induced in dose-dependent manner (Figure 2A). Stable expression of LMP1 also increased the level of Snail protein (Figure 2B) and mRNA (Figure 2C).

### LMP1 induces EMT phenotype in NP69SV40T human nasopharyngeal cells

We previously found that LMP1 could induce EMT in the canine cell line MDCK, and Twist has an important role in this context (Horikawa *et al*, 2007). Snail was not upregulated by LMP1 and was not involved in LMP1-induced EMT in MDCK cells (data not shown). However, the present analyses of NPC tissues suggested that Snail might be more closely involved with EMT in NPC than Twist. Therefore, we extended our studies of involvement of these EMT regulators by exploiting a new human cell-culture model. Madin-Darby Canine-Kidney cells are widely used as the model cells for study of EMT (Horikawa *et al*, 2007; Thiery *et al*, 2009), but they are canine-kidney cells and may not be best suited for study of a human virus. We have been using human AdAH nasopharyngeal cells in a series of studies of LMP1's role in metastasis, but in AdAH cells, LMP1 produced neither morphological change nor EMT-like features (Yoshizaki *et al*, 2005; Horikawa *et al*, 2007).

**Figure 3:**
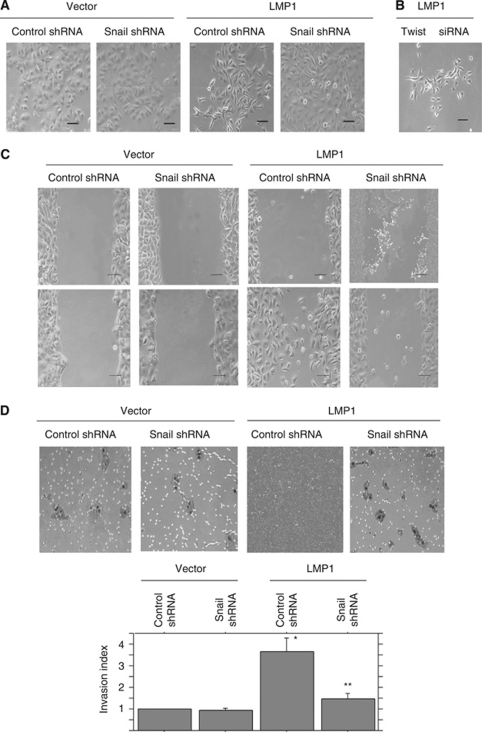
LMP1 induces EMT phenotype through Snail in a human nasopharyngeal cell line. (**A**) Morphological changes in NP69SV40T nasopharyngeal epithelial cells induced by transfection with LMP1 could be reversed by Snail shRNA. Snail shRNA and control shRNA were transduced by the retroviral system to generate stable clones. NP69SV40T cell clones, 2 × 10^5^ cells per dish, were plated onto 35-mm plastic dishes and observed after 24 h. Representative clones are shown. Bars, 50 *μ*m. (**B**) Transduction of Twist siRNA in LMP1-transfected NP69SV40T cells did not change the cellular phenotype. (**C**) Scrape-wound migration assay shows that enhanced motility in LMP1-transfected cells is downregulated by silencing Snail through Snail shRNA. Confluent monolayers of NP69SV40T nasopharyngeal epithelial cell clones were scraped with a plastic pipette tip, and migration of cells was observed. Typical wounds at 0 and 10 h are shown. Bars, 200 *μ*m. (**D**) Enhanced invasiveness of LMP1-transformed cells is downregulated by Snail shRNA. After 72 h in the Matrigel invasion assays, each NP69SV40T cell clone adherent on the lower surface of the filter was fixed and stained. Representative photographs are shown. Bar, 50 *μ*m. Invasion indices were calculated from the counts of cells invading through Matrigel-coated membrane. Significance was tested by paired *t*-test. ^*^P=0.0007 as compared with control cells (NP69SV40T+vector+control shRNA cells); ^**^P=0.0041 as compared with NP69SV40T+LMP1+control shRNA cells.

An immortalised human nasopharyngeal epithelial cell line, NP69SV40T, was derived recently from primary non-malignant nasopharyngeal epithelial cells (Tsao *et al*, 2002). It is highly responsive to LMP1 and offers a new cell system for study of the role of EBV in NPC (Tsao *et al*, 2002; Lo *et al*, 2006). First, we tested whether EBV LMP1 could also induce EMT in this more relevant cell system. Parental NP69SV40T and cells stably transfected with a control plasmid exhibited typical polygonal epithelial cell morphology and grew in a cohesive pattern (Figure 3A). By contrast, LMP1 transfectants acquired an elongated fibroblast-like appearance and grew in a scattered pattern with reduction in cell-to-cell contacts (Figure 3A). These changes represent hallmarks of EMT.

### Snail, but not Twist, is important for induction of EMT, cell migration and invasiveness by LMP1 in human nasopharyngeal cells

**Figure 4:**
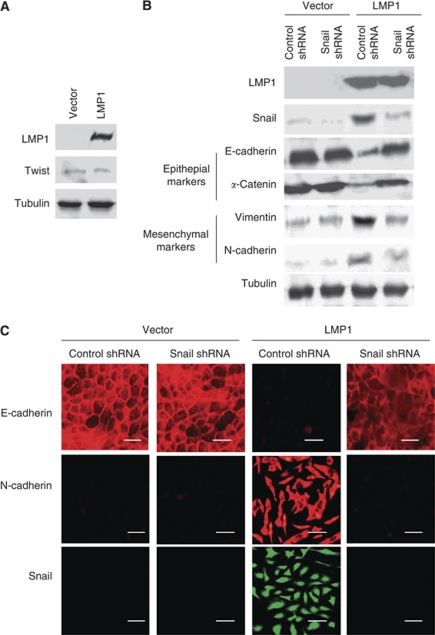
Molecular markers confirm that LMP1 induces EMT through Snail in NP69SV40T nasopharyngeal cells. (**A**) Transfection of LMP1 does not change levels of Twist in NP69SV40T cells. (**B**) Expression of representative epithelial markers, E-cadherin and *α*-catenin, and mesenchymal markers, vimentin and N-cadherin, together with LMP1 and Snail in NP69SV40T cell clones is shown by western blotting. (**C**) Immunofluorescence staining for a representative epithelial marker, E-cadherin, and mesenchymal marker, N-cadherin, in NP69SV40T cell clones together with staining for Snail are shown. Bars, 20 *μ*m.

We examined expression of Twist in NP69SV40T cells. Twist was detected in trace amounts in these cells. In contrast to MDCK cells, levels of Twist were not upregulated by LMP1 (Figure 4A). Neither SIP1 nor Slug was detected in clones of NP69SV40T cells. We next explored involvement of Snail in LMP1-induced EMT in this nasopharyngeal cell line. Expression of Snail was clearly upregulated by LMP1 in NP69SV40T cells (Figure 4B). Moreover, silencing of Snail expression by Snail small hairpin RNA (shRNA) in LMP1-transfected NP69SV40T cells largely reversed the cellular phenotype to an epithelial morphology (Figure 3A). Transduction of Twist siRNA in LMP1-transfected NP69SV40T cells did not change the cellular phenotype (Figure 3B).

In a wound-induced cell-migration assay, LMP1-transfected NP69SV40T cells migrated into the wounded area away from the monolayer edges (Figure 3C). Suppression of expression of Snail expression with shRNA clearly reduced such cellular motility (Figure 3C). Also, in a Matrigel cell-invasion system, LMP1-induced invasiveness was significantly inhibited by Snail shRNA (Figure 3D). Thus, Snail, but not Twist, is critical for causing LMP1-induced EMT and invasive properties in these human nasopharyngeal cells.

### Molecular markers confirmed the essential role of Snail in LMP1-induced EMT

In addition, we examined levels of epithelial and mesenchymal markers in NP69SV40T cells. Upon transfection with LMP1, the level of representative epithelial cell markers decreased, whereas the expression of mesenchymal markers increased markedly (Figure 4B). Immunofluorescence studies confirmed the disappearance of E-cadherin from cell membranes as well as the induction of N-cadherin in the cytoplasm caused by LMP1 (Figure 4C). Hence, molecular changes confirmed that LMP1 induces EMT in human nasopharyngeal cells. Further, in the Snail shRNA-transfectants, changes in expression of the molecular markers were consistent with mesenchymal-to-epithelial transition (MET), the reverse of EMT (Yang and Weinberg, 2008; Thiery *et al*, 2009). This result fortified the conclusion that EBV LMP1 can induce EMT in NPC as well as underscore the principal role of Snail in this process.

## Discussion

Epithelial–mesenchymal transition has recently been proposed as a major metastasis-promoting mechanism and implicated in several types of invasive human malignancies (Yang and Weinberg, 2008; Thiery *et al*, 2009). The genesis of EMT and its oncogenic role in carcinomas are beginning to be clarified. We reported earlier the novel finding that EMT is induced by LMPI, the EBV oncoprotein that is a member of the TNFR super-family, and contributes to metastatic features of NPC (Horikawa *et al*, 2007). Recent studies of EMT are generating a major shift in thinking in the field of cancer metastasis (Ansieau *et al*, 2008; Klein, 2008; Mani *et al*, 2008; Sánchez-García, 2009). Metastasis has generally been considered to result from the accumulation of genetic and epigenetic changes in the primary tumour as a final step in oncogenesis. This long-held view is increasingly challenged by a new early metastasis model operating through EMT, which postulates that metastasis occurs beginning at an early phase in tumour development (Ansieau *et al*, 2008; Klein, 2008; Mani *et al*, 2008; Sánchez-García, 2009). Nasopharyngeal carcinoma, a carcinoma characterized by its proclivity to invade and metastasise early, has several EMT-like features (Lo *et al*, 2004; Wei and Sham, 2005). Therefore, in this study, we have again focused on NPC as a biologically useful model for study of EMT and metastasis.

We report here that the EMT regulator, Snail, as well as Twist are overexpressed in NPC and that expression of LMP1 and Snail correlated with each other in NPC. Moreover, expression of Snail correlated independently inversely with expression of E-cadherin, and was associated with metastasis in NPC. By contrast, expression of Twist had no association with expression of E-cadherin. Further, in the human nasopharyngeal epithelial cell line, NP69SV40T, LMP1 could induce EMT, and Snail, but not Twist, played a pivotal role in this context. Nasopharyngeal carcinoma stands out for its highly metastatic character and undifferentiated histopathology among head-and-neck carcinomas (Lo *et al*, 2004; Wei and Sham, 2005). This study strengthens our view that the genesis of EMT by EBV LMP1 contributes to the distinctive early metastatic character of NPC. These findings also raise the possibility that NPC offers a clinical model for EMT. Intriguingly, and differing from our earlier finding in canine cells, this study newly suggests Snail as the pivotal EMT regulator in NPC.

Several EMT regulators show varied expression and roles in different kinds of cell lines and in different human carcinomas. Different patterns of expression of Snail, SIP1 and Twist according to histological subtype are reported for gastric cancer (Rosivatz *et al*, 2002). In breast cancer, Twist and Slug are upregulated and associated with progression, whereas Snail is downregulated (Martin *et al*, 2005). Slug expression is associated with downregulation of E-cadherin in diffuse and intestinal-type gastric carcinoma, and this effect is complemented by SIP1 and Snail (Castro Alves *et al*, 2007). Studies of prostate cancer have revealed that Snail and Slug lead to a reduction of E-cadherin expression, and Twist leads to a further decrease (Alexander *et al*, 2006). In our study, the levels of SIP1 and Slug were marginal and had no association with clinical parameters in NPC, whereas Snail and Twist were clearly overexpressed and showed significant positive correlation with metastasis. Moreover, Snail by itself, but not Twist, inversely correlated with E-cadherin, which suggests Snail's dominant role in the progression of EMT in NPC. Previously, we reported the key role of Twist in EMT in MDCK cells (Horikawa *et al*, 2007). Here, we have identified the central role of Snail in EMT in NP69SV40T human nasopharyngeal cells. The discrepancy may be attributed to differences in cell species and perhaps also cell type. MDCK cells are the first and most used model cell system for the study of EMT (Lo *et al*, 2004), but it is literally a cell line of canine-kidney origin. The immortalised human nasopharyngeal epithelial cell line, NP69SV40T, retains many characteristics of normal nasopharyngeal cells and is highly responsive to EBV LMP1 (Tsao *et al*, 2002). As LMPI is expressed in earliest stages of the genesis of NPC (Raab-Traub, 2002; Pagano *et al*, 2004; Pagano, 2009), we view these cells as offering the first credible cell-culture model for premalignant nasopharyngeal epithelial cells. The behaviour of Snail in these nasopharyngeal cells underscores the prime role of Snail in EMT in relation to NPC, but Twist may also have functions in this context. Its overexpression is reported to induce angiogenesis and chromosomal instability *in vivo* (Mironchik *et al*, 2005), and Twist inhibits both p53-dependent and p53-independent apoptosis and favors cell survival (Puisieux *et al*, 2006). The mechanisms of EMT and metastasis in NPC are undoubtedly diverse, but the current results clearly point to a pivotal role for Snail in EMT and metastasis of NPC. This study lends substantial credibility to the importance of EMT in metastasis of human carcinoma. Investigation of EMT in NPC is likely to continue to illuminate the mechanisms of metastasis of NPC and an array of human carcinomas.
